# Red Rubiscos and opportunities for engineering green plants

**DOI:** 10.1093/jxb/erac349

**Published:** 2022-09-02

**Authors:** Zhen Guo Oh, Bryce Askey, Laura H Gunn

**Affiliations:** Plant Biology Section, School of Integrative Plant Science, Cornell University, Ithaca, NY, USA; Plant Biology Section, School of Integrative Plant Science, Cornell University, Ithaca, NY, USA; Plant Biology Section, School of Integrative Plant Science, Cornell University, Ithaca, NY, USA; Department of Cell and Molecular Biology, Uppsala University, S-751 24 Uppsala, Sweden; Lancaster University, UK

**Keywords:** Carboxylation, photosynthesis, plastome, protein engineering, Rubisco, structure–function

## Abstract

Nature’s vital, but notoriously inefficient, CO_2_-fixing enzyme Rubisco often limits the growth of photosynthetic organisms including crop species. Form I Rubiscos comprise eight catalytic large subunits and eight auxiliary small subunits and can be classified into two distinct lineages—‘red’ and ‘green’. While red-type Rubiscos (Form IC and ID) are found in rhodophytes, their secondary symbionts, and certain proteobacteria, green-type Rubiscos (Form IA and IB) exist in terrestrial plants, chlorophytes, cyanobacteria, and other proteobacteria. Eukaryotic red-type Rubiscos exhibit desirable kinetic properties, namely high specificity and high catalytic efficiency, with certain isoforms outperforming green-type Rubiscos. However, it is not yet possible to functionally express a high-performing red-type Rubisco in chloroplasts to boost photosynthetic carbon assimilation in green plants. Understanding the molecular and evolutionary basis for divergence between red- and green-type Rubiscos could help us to harness the superior CO_2_-fixing power of red-type Rubiscos. Here we review our current understanding about red-type Rubisco distribution, biogenesis, and sequence–structure, and present opportunities and challenges for utilizing red-type Rubisco kinetics towards crop improvements.

## Teaser

We present a review of red- and green-type Rubisco functional and regulatory divergence, and how these present both challenges and opportunities to boost photosynthetic carbon assimilation in agricultural plants.

## Introduction

Rubisco represents the major point of carbon entry into the biosphere, catalysing the addition of a CO_2_ molecule to the five-carbon sugar, ribulose-1,5-bisphosphate (RuBP) (Knight *et al.*, 1990; Cleland *et al.*, 1998). For productive substrate binding, Rubisco must be first activated by priming a strictly conserved catalytic lysine with a non-substrate CO_2_ molecule, which is subsequently stabilized by a magnesium ion (Lorimer and Miziorko, 1980). A series of complex partial reactions ultimately yields two molecules of 3-phosphoglycerate that are fed into the Calvin–Benson–Bassham (CBB) cycle for carbohydrate production (Calvin and Benson, 1948). Rubisco catalysis is notoriously inefficient, exhibiting both a slow catalytic turnover rate (*k*_cat, C_) and a limited ability to discriminate between CO_2_ and O_2_, as quantified by its specificity factor (*S*_C/O_). Recycling the byproduct of RuBP oxygenation via photorespiration comes at the cost of energy and release of a previously fixed CO_2_ (Bauwe *et al.*, 2010). Such slow and promiscuous catalysis means that Rubisco limits the efficiency of light-saturated photosynthesis in the leaves of plants (Long *et al.*, 2006). Many species compensate for poor kinetic performance by producing large quantities of Rubisco, representing a large nitrogen investment (Ellis, 1979). However, some organisms have independently evolved CO_2_-concentrating mechanisms (CCMs) that limit oxygenation by elevating CO_2_ concentrations at Rubisco active sites. These include biochemical CCMs such as C_4_ and crassulacean acid metabolism (CAM) photosynthesis, and biophysical CCMs such as carboxysomes and pyrenoids (for a review, see Meyer and Griffiths, 2013).

Rubiscos can be classified into three carboxylation-competent forms, Forms I–III (Tabita *et al.*, 2008b), which exhibit divergent sequence and structure. All Rubisco forms probably derive from a common Form III ancestor, which was transferred via lateral gene transfer to a common ancestor of proteobacteria and cyanobacteria (Tabita *et al.*, 2008a). The Form III Rubiscos distributed in archaea scavenge toxic byproducts of metabolism (Sato *et al.*, 2007), while Form II Rubiscos in proteobacteria and dinoflagellates (Rowan *et al.*, 1996; Badger and Bek, 2008) operate in the CBB cycle. Form I Rubiscos are the most abundant Rubisco form and comprise eight ~50–52 kDa large subunits (LSus; *rbcL* or *cbbL* gene) and eight ~15 kDa small subunits (SSus; *RbcS* or *cbbS* gene). Two active sites are formed at the interface of two LSus within an L_2_ Rubisco dimer. Four L_2_ dimers form an octameric L_8_ core, which is then capped at each end by two SSu tetrads forming the ~550 kDa L_8_S_8_ holoenzyme (Knight *et al.*, 1990).

Significant variation in Form I Rubisco catalysis exists in nature, with Rubisco variants from non-green algae possessing superior kinetic properties that could boost carbon assimilation in chloroplasts (Whitney *et al.*, 2001; Zhu *et al.*, 2004). This sequence-distinguishable lineage of Rubiscos are often called ‘red-type’ Rubiscos and are found in rhodophytes (Form ID), their symbionts: cryptophytes, haptophytes, and heterokonts (Form ID), and certain proteobacteria (Form IC), whereas ‘green-type’ Rubiscos are distributed in terrestrial plants, chlorophytes (Form IB), cyanobacteria (Form IA/IB), and some proteobacteria (Form IA) (Delwiche and Palmer, 1996). Red-type, especially Form ID, Rubiscos break the canonical catalytic trade-off between *k*_cat, C_ and *S*_C/O_ observed for green-type Rubiscos (Young *et al.*, 2016)—a trend previously used to justify claims that Rubisco catalysis has reached an evolutionary maximum and thus its kinetics cannot be further improved (Tcherkez *et al.*, 2006). The extent of, and driving force behind, these trade-offs have recently been investigated (Flamholz *et al.*, 2019; Bouvier *et al.*, 2021; Tcherkez and Farquhar, 2021), and red-type Rubiscos show that any catalytic trade-offs are not universal, providing optimism that it may be possible to engineer catalytically enhanced crop Rubiscos.

Prior reviews and commentaries have provided detailed descriptions of specific aspects of red-type Rubisco functional divergence (Hanson, 2016; Raven and Giordano, 2017). Iñiguez *et al.* (2020) and Rickaby and Hubbard (2019) provide insightful interpretations of red-type Rubisco kinetic variation, especially in the context of environmental constraints. Discussions about SSu-mediated Form IC Rubisco biogenesis (Hauser *et al.*, 2015) and the functional divergence of the accessory proteins that maintain activated red- and green-type Rubisco pools (Bhat *et al.*, 2017) are especially useful to appreciate the requirements for optimal red-type Rubisco function in heterologous systems. Phylogenetic relationships between the red- and green-type Rubisco lineages have also been reviewed extensively (Tabita *et al.*, 2007, 2008a, b; Liu *et al.*, 2017). This review is aimed towards a holistic understanding as to how differences in evolutionary history, sequence–structure–function, biogenesis and modulation between the red- and green-type Rubisco lineages present opportunities and/or challenges to confer red-like kinetics to green plants for increased crop production.

## The distribution of red-type Rubiscos and red plastids

**Fig. 1.**
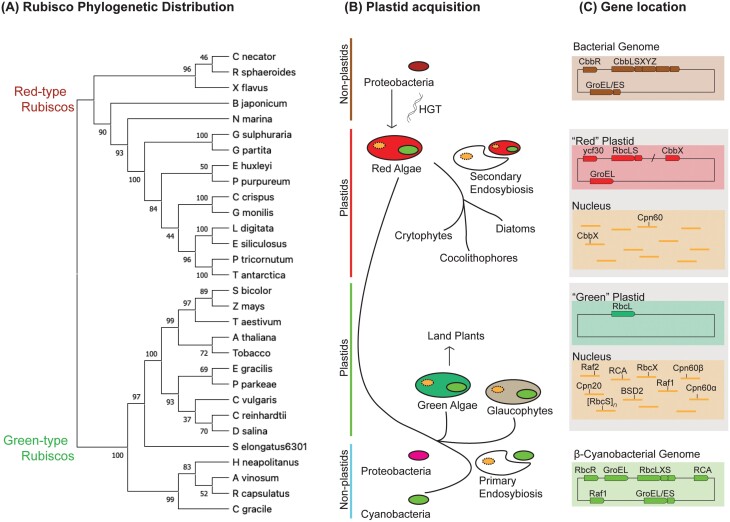
Red- and green-type Rubisco distribution, plastid lineage, and gene locations. (A) LSu phylogenetic tree showing a clear distinction between the red- and green-type Rubisco lineages. Notably, Rubisco distribution is inconsistent with organismal relationships, with prokaryotic and eukaryotic autotrophs clustering together within each of the red and green lineages. This is consistent with horizontal gene transfer of Rubisco-encoding genes from the proteobacteria to the ancestor of red plastids. Phylogenetic analyses were performed in MEGA11 (Stecher *et al.*, 2020; Tamura *et al.*, 2021), using the Maximum Likelihood method and JTT matrix-based model (Jones *et al.*, 1992), with elimination of all positions containing gaps. The percentages of replicate trees in which the associated taxa clustered together in the bootstrap test (1000 replicates) are shown next to the branches (Felsenstein, 1985). Phylogenetic clusters of proteobacterial Form IC, eukaryotic Form ID, eukaryotic Form IB, and proteobacterial and cyanobacterial Form IA and Form IB Rubiscos are indicated by brown, red, green, and turquoise bars, respectively. (B) Plastid acquisition events that lead to the red and green plastid lineages. Primary endosymbiosis, where a cyanobacterium was engulfed by a eukaryotic host cell, led to the formation of three major extant clades, namely glaucophytes, chlorophytes (green algae), and rhodophytes (red algae). Green algae eventually give rise to land plants, while secondary red lineage symbionts such as cryptophytes, coccolithophores, and diatoms arose from endosymbiosis of a red alga. Red algal Rubisco-encoding genes were acquired by a horizontal gene transfer (HGT) event from a proteobacterium. Branch lengths are not to scale. (C) Generalized genome locations of Rubisco-related genes for bacterial, red lineage, green lineage, and β-cyanobacterial genomes. CbbLSXYZ, GroEL/ES, RbcLS/CbbX, and RbcLXS (RbcL–RbcX–RbcS) operons are found in bacterial, β-cyanobacterial, and red-plastid genomes. However, CbbX in red plastids is not always found downstream of the RbcLS operon. CbbR, RbcR, and ycf30 are generally located upstream of Rubisco-encoding genes. Only the Rubisco LSu-encoding gene remains in the green plastid genome, while all other known Rubisco-related genes are located in the nucleus, including genes involved in Rubisco biogenesis (Cpn60α, Cpn60β, Cpn20, Raf1, Raf2, RbcX, and BSD2) and reactivation of inhibited Rubisco (RCA). These green nuclear genomes also encode multiple copies of RbcS. Note that Raf1, RbcX, and RCA are not always found in β-cyanobacterial genomes and glaucophyte gene arrangement(s) are excluded from this figure. Accession codes used for construction of the phylogenetic tree: *Griffithsia monilis* (ABU53651.1), *Chondrus crispus* (M5DDJ6), *Galdieria partita* (IBWV_A), *Galdieria sulphuraria* (AIG92599.1), *Porphyridium purpureum* (BAO23622.1), *Thalassiosira antarctica* (5MZ2_A), *Ectocarpus siliculosus* (P24313), *Phaeodactylum tricornutum* (ABK20641.1), *Laminaria digitata* (AGM75436.1), *Emiliana huxleyi* (Q4G3F4), *Rhodobacter sphaeroides* (5NV3_A), *Xanthobacter flavus* (P23011.1), *Nitrosomonas marina* (A0A1I0FH32), *Cupriavidus necator* (CAJ96184.1), *Bradyrhizobium japonicum* (GEC50337.1), *Arabidopsis thaliana* (5IU0_A), tobacco (NP_054507.1), *Triticum aestivum* (QBK83209.1), *Sorghum bicolor* (ABK79504.1), *Zea mays* (NP_043033.1), *Chlamydomonas reinhardtii* (1GK8_A), *Chlorella vulgaris* (NP_045897.1), *Euglena gracilis* (NP_041936.1), *Dunaliella salina* (ACS95083.1), *Pyramimonas parkeae* (ACJ71114.1), *Synechococcus elongatus* PCC 6301 (1RSC_A), *Cyanobium gracile* (K9P2B9), *Halothiobacillus neapolitanus* (1SVD_A), *Rhodobacter capsulatus* (AAC37141.1), and *Allochromatium vinosum*, (AAA23328.1).

The first plastid arose ~1.5 billion years ago via a primary endosymbiotic event where a eukaryotic cell engulfed a free-living cyanobacterium (for a review, see McFadden, 2001), before divergence into *Glaucocystophyta* (microalgae), *Chlorophyta* (green algae), and *Rhodophyta* (red algae) plastid lineages (Fig. 1B). Subsequent endosymbiosis of chlorophytes and rhodophytes gave rise to the plethora of plastid lineages presently observed in photosynthetic eukaryotes. This monophyletic origin of plastids (Delwiche and Palmer, 1996; Delwiche, 1999) is supported by the organismal relationships observed in phylogenetic trees constructed using non-Rubisco-encoding genes, where rhodophytes and chlorophytes cluster distinctively from cyanobacteria and proteobacteria. However, LSu-based phylogenies demonstrate a clear distinction between red- (Form IC and ID) and green- (Form IA and IB) lineage Rubiscos (Delwiche and Palmer, 1996) (Fig. 1A). This is observed as chlorophyte Rubiscos were acquired during endosymbiosis, while Rubisco-encoding genes in eukaryotic red-type organisms were acquired from a proteobacterium through horizontal gene transfer before the secondary endosymbiotic events (Iñiguez *et al.*, 2020). Nonetheless, Rubisco LSus are highly conserved, exhibiting ~80% amino acid identity within red and green lineages, and ~60% across lineages (Parry *et al.*, 2003). Divergence in SSu sequences is more apparent, with ~50–60% sequence identity within each of the red and green lineages, but only ~30% identity observed across groups.

## Red and green plastid environments provide opportunities for divergent evolution

Perhaps the most characteristic difference in plastid environments is that while rhodophytes have Chl *a* and phycobiliproteins, chlorophytes contain Chl *a* and Chl *b* (Delwiche, 1999; Cavalier-Smith, 2000). Secondary symbionts of red algae have Chl *a*, Chl *c*, and either phycobiliproteins or fucoxanthin (Falkowski *et al.*, 2004). These pigment differences are the reason for use of the terms ‘red’ and ‘green’ when describing different plastid lineages and, by extension, the Rubisco they express. Plastid architecture distinctions are also present, with green lineage plastids containing stacked thylakoids. Red algal thylakoids are unstacked, but may be stacked in some of their secondary symbionts (Bisalputra and Bailey, 1973; Ford, 1984; Flori *et al.*, 2017; Arshad *et al.*, 2021).

After the divergence of the red and green lineage plastids, organisms underwent massive (30- to 40-fold) plastome reduction events, where protein-encoding genes in the plastome were lost because of functional redundancy or were transferred to the nuclear genome (Delwiche, 1999; Archibald and Keeling, 2002; Stegemann *et al.*, 2003; Timmis *et al.*, 2004). Rhodophytes underwent substantially less plastome reduction and their plastomes presently contain roughly twice as many protein-coding genes as chlorophytes (Ohyama *et al.*, 1986; Shinozaki *et al.*, 1986; Reith and Munholland, 1995). Thus, there is a difference in the genes that could potentially co-evolve in the red and green plastomes. A key example is that in rhodophytes, Rubisco- and putative chaperone-encoding genes were retained in the plastome and are co-transcribed as part of an operon, whereas in chlorophytes, the genes encoding the Rubisco SSu and many chaperones were transferred to the nucleus (Zauner *et al.*, 2006) (Fig. 1C).

Gene duplication events in chlorophytes led to a nuclear-encoded SSu multigene family, where distinct SSu isoforms are differentially expressed. As SSu content indirectly controls LSu synthesis and total L_8_S_8_ pools (see Wietrzynski *et al.*, 2021), differential SSu expression allows total Rubisco pools in chlorophytes to vary in response to environmental cues (Yoon *et al.*, 2001; Khumsupan *et al.*, 2020), and may also confer kinetic variability to the holoenzyme (Lin *et al.*, 2020). Coding of green-type Rubisco genes in distinct subcellular locations necessitates an N-terminal transit peptide on SSu-encoding genes to target them to the chloroplast (Razzak *et al.*, 2017), and bi-directional crosstalk between the plastid and nucleus to coordinate LSu and SSu expression (Nott *et al.*, 2006; Koussevitzky *et al.*, 2007; Zhang, 2007). In contrast, rhodophyte Rubisco LSu and SSu stoichiometry is maintained by the coupled transcription inherent to their operon arrangement, which may have allowed rhodophytes to avoid potential sequence–space limitations associated with coordinating LSu and SSu expression. Despite the possible constraints on green-type Rubisco evolution, the chlorophyte system may provide opportunities for more dynamic control of total Rubisco content and activity in chloroplasts. In addition to the plastome operon, the absence of introns and sequence repeats in red lineage plastomes (Oudet-Le-Secq *et al.*, 2007) are consistent with eukaryotic red-type Rubiscos being acquired from a proteobacterium (Delwiche and Palmer, 1996).

## Red-type Rubisco structural divergence

### Red and green LSus: the same, but different

**Fig. 2.**
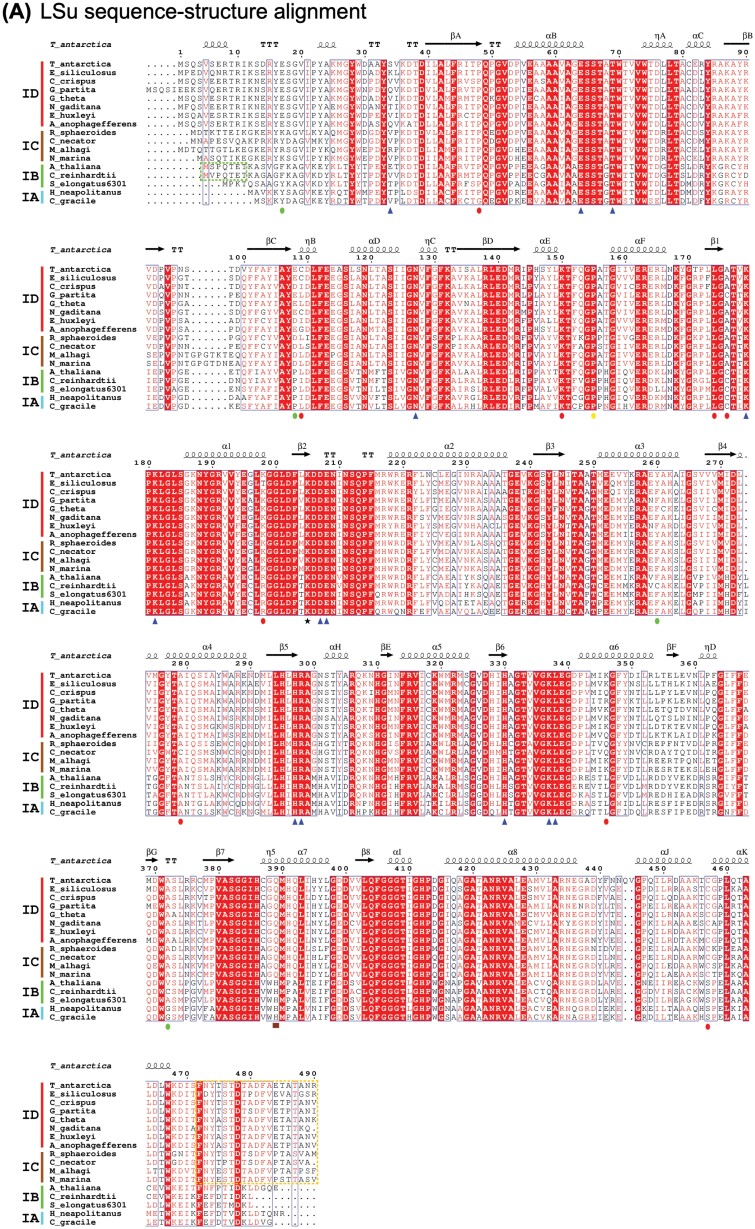

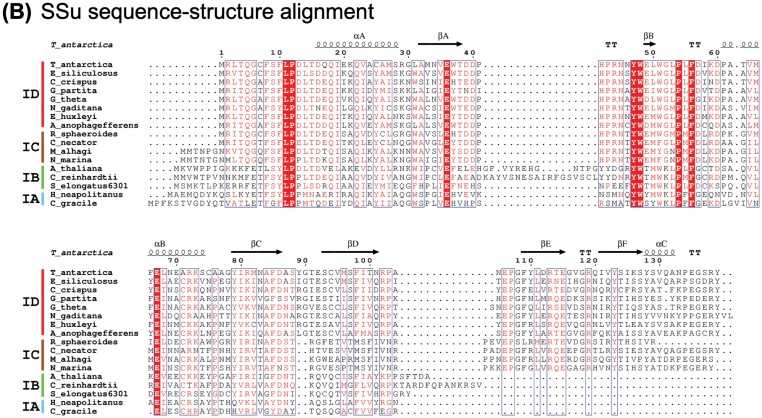
Sequence–structure alignment of red- and green-type Rubiscos. Structure-based sequence alignments are shown for (A) LSus and (B) SSus from eight Form ID (red algae, diatoms, cryptophytes, haptophytes, and pelagophytes), four Form IC proteobacteria, three Form IB (vascular plant, green alga, and β-cyanobacteria), and two Form IA (proteobacteria and α-cyanobacterial) Rubiscos. Secondary structural features of Rubisco SSus are labelled according to convention (Knight *et al.*, 1990). Residue numbering for sequences and structural annotations (α, α-helix; η, 310-helix; β, β-strand; TT, tight β-turns) are relative to *Thalassiosira antarctica* Rubisco (5MZ2). Symbols at the bottom of sequences indicate residues contributing to the active site (blue triangles), the catalytic lysine residue (black star), and the red-type Rubisco latch residue (brown square). Post-translational modifications found in green-type (green circles), red-type (red circles), or both (yellow circle) are also indicated. Conserved N-terminal residues in Form IB eukaryotic Rubiscos and C-terminal residues in Form ID and IC Rubiscos that are threaded by Rubisco activase RCA and CbbX are marked with a green and orange dotted box, respectively. The alignment was created using the Rubisco accession numbers (LSu, SSu): *T. antarctica*, (5MZ2_A, 5MZ2_I), *E. siliculosus* (P24313, P24395), *C. crispus* (M5DDJ6, M5DD36), *G. partita* (1BWV_A, 1BWV_B), *G. theta* (P14957, P14957), *N. gaditana* (K9ZV74, A0A023PJK0), *E. huxleyi* (Q4G3F4, Q4G3F3), *A. anophagefferens* (C6KIP8, C6KIP9), *R. sphaeroides* (5NV3_A, 5NV3_B), *C. necator* (1BXN_A, 1BXN_B), *M. alhagi* (H0HRD0, H0HRD1), *N. marina* (A0A1I0FH32, A0A1I0FH45), *A. thaliana* (5IU0_A, 5IU0_C), *C. reinhardtii* (1GK8_A, 1GK8_E), *S. elongatus* PCC 6301 (1RSC_A, 1RSC_B), *H. neapolitanus* (1SVD_A, 1SVD_B), and *C. gracile* (K9P2B9, K9P3U4). Alignments were performed using T-coffee (Expresso mode) (Di Tommaso *et al.*, 2011), followed by manual curation of output files. Graphics were generated with ESPript (Robert and Gouet, 2014).

**Fig. 3.**
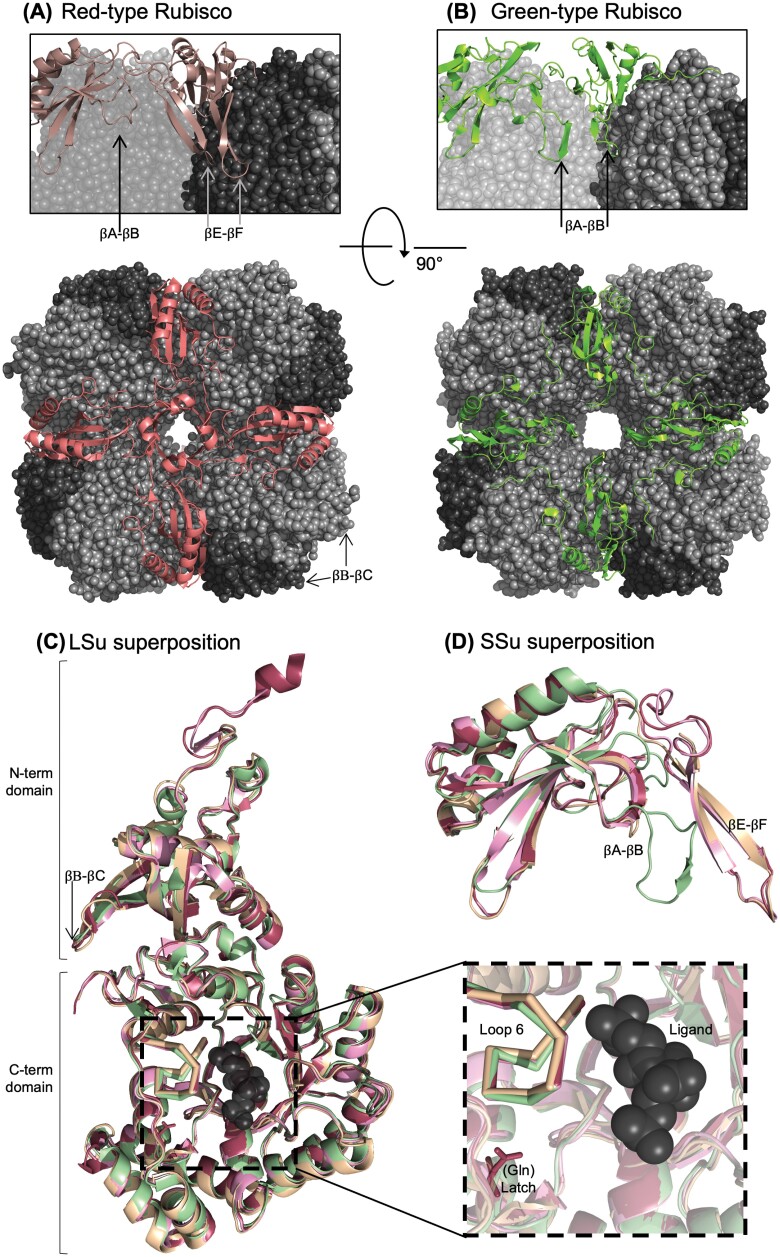
Key structural differences between red- and green-type Rubiscos. Top view of the (A) red-type Rubisco from *Thalassiosira antarctica*, and (B) green-type Rubisco from *Arabidopsis thaliana*, showing differences in SSu (green/red) loops packing against LSu cores (in greys). Inset views show the respective hairpin structures at the interface of two LSu dimers. Location of sequence insertions in the βB–βC loops of certain proteobacterial red Rubiscos are indicated. Superpositions of Rubisco (C) LSus and (D) SSus, with subunits from a diatom (*T. antarctica*), red alga (*Galdieria partita*), proteobacterial red-type (*Rhodobacter sphaeroides*), and green-type (*A. thaliana*) Rubisco shown in red, pink, yellow, and green, respectively. In (C), the inset highlights ligand bound at the active site (spheres), the catalytic loop 6 (ribbon), and the red latch residue (stick). Images were created in the PyMOL Molecular Graphics System (v.1.7.4, Schrödinger), using the PDB coordinates 5IU0, 5MZ2, 1BWV, and 5NV3 for Rubisco from *A. thaliana*, *T. antarctica*, *G. partita*, and *R. sphaeroides*, respectively.

The Rubisco LSu N-terminal domain comprises a four-stranded β-sheet and two α-helices, and the C-terminal domain forms a barrel containing eight βα units. The active site is formed by four residues from the N-terminal domain and six residues within the C-terminal domain of the adjacent LSu (Andersson and Backlund, 2008; Kannappan and Gready, 2008) (Fig. 2A). During catalysis, loop 6 closes over the active site, and is stabilized by interactions with LSu C-tail residues. Red-type Rubisco LSus exhibit structural divergence from other Rubisco lineages. When loop 6 in red-type Rubisco is closed (i.e. Rubisco is in the closed state), a latch structure is formed by a H-bond between a highly conserved valine residue at the start of loop 6 and a glutamine residue in helix α7 (Okano *et al.*, 2002) (Fig. 2A). Modifying the highly conserved histidine in green-type Rubiscos at this position to glutamine enhances *S*_C/O_ (Ninomiya *et al.*, 2008). Further sequence–structural variation is found in certain Form IC Rubiscos, where a six amino acid insertion is found in the solvent-exposed βB–βC loop in the LSu N-terminal domain (Utåker *et al.*, 2002; Umezawa *et al.*, 2016) (Fig. 3A, B, and see Fig. 2A). The potential functional influence of these loops is not known. This could be investigated by modifying these βB–βC loops via site-directed mutagenesis, and comparing characteristics such as kinetic performance, folding capacity, and thermostability between heterogolously produced wild-type and modified Rubiscos.

### The red-type Rubisco SSu exhibits distinctive structure–function divergence

The canonical Rubisco SSu core structure consists of four-stranded antiparallel β-sheets and two α-helices. Despite not contributing residues to, and being spatially separated from, the active site, the SSu exerts a catalytic influence on the Rubisco holoenzyme presumably via some long-range communication (van Lun *et al.*, 2011). Alternatively, molecular dynamics simulations suggest that the SSu could be important for channelling CO_2_ to the active site (van Lun *et al.*, 2014). This SSu catalytic influence has been demonstrated by a large number of studies using chimeric (Spreitzer *et al.*, 2001, 2005; Spreitzer, 2003; Karkehabadi *et al.*, 2005; Genkov *et al.*, 2010) or hybrid (Read and Tabita, 1992; Wang *et al.*, 2001; Genkov and Spreitzer, 2009; Ishikawa *et al.*, 2011; Zhang *et al.*, 2011; Morita *et al.*, 2014) Rubiscos. Red-type Rubisco SSus have a slightly shorter βA–βB loop, longer βC–βD loop, and shorter N-terminus compared with green-type Rubiscos (Fig. 2B). However, green- and red-type SSus are readily distinguishable by one major structural feature present in only red-type Rubisco SSus—two extra β-sheets at the C-terminus known as the βE–βF hairpins (Figs 2B, 3A, D). Despite being formed from different structural regions within the SSu, the βE–βF hairpins are considered to be equivalent to the green-type SSu βA–βB loops in terms of their relative position within the holoenzyme quaternary structure, lining the central solvent channel (Fig. 3A, B). The red-type βE–βF hairpins form more extensive contacts with LSu residues in the central pore than their green counterparts (Hansen *et al.*, 1999; Sugawara *et al.*, 1999). More extensive H-bonding at Rubisco interfaces correlates with enhanced CO_2_ specificity (van Lun *et al.*, 2011). In addition to a pervasive influence on *S*_C/O_ (Spreitzer, 2003; Spreitzer *et al.*, 2005; van Lun *et al.*, 2011; Joshi *et al.*, 2015), the βE–βF hairpins also play a role in Rubisco biogenesis. Transplanting the *Rhodobacter sphaeroides* (red-type) βE–βF hairpin sequence into *Synechococcus* PCC 6301 (green-type) Rubisco circumvents the assembly requirement for RbcX (Joshi *et al.*, 2015), a molecular chaperone absent from species with red-type Rubiscos.

### Past and future insights from structural data

Structural models of Rubisco from eight red-type and 10 green-type species are available in the Protein Data Bank (PDB). Apart from the previously discussed structural variation, these Rubiscos exhibit strikingly similar structure. Minor structural alterations and small sample size cloud our understanding of additional catalysis-influencing structural variance between the red and green lineages, and especially between higher and lower performing red-type variants (i.e. those that exhibit higher/lower carboxylation efficiencies). Few structures have been published of Rubisco in the open conformation (PDB: 1AUS, 3AXK, and 7JN4), as a ligand is often bound to Rubisco to stabilize flexible loops, allowing for tighter crystal packing or to reduce structural heterogeneity for cryoEM. Recent advances in the ability to resolve distinct structural states *in silico* using cryoEM (Punjani and Fleet, 2021) may lead to an increase in the number of Rubisco structures in the open conformation and provide insight into dynamic differences between red- and green-type Rubiscos.

CryoEM structures of Rubisco LSus in complex with CCM components (PDB: 7JFO and 6HBC), molecular chaperones (PDB: 7VWX, 3ZQ1, 3ZPZ, 6LRR, 6SMH, 2WVW, 6Z1F, and 6Z1G), and an L_8_S_4_ assembly intermediate (PDB: 6LRS) have recently become available. The single-particle nature of cryoEM, and increasing capabilities to resolve structural heterogeneity within datasets, provides a distinct advantage to capture more transient and low occupancy interactions than X-ray crystallography. Similar attention to red-type Rubisco subunits and their interaction with known or putative interacting proteins during Rubisco biogenesis could provide insights into potential mechanistic and biogenesis differences between the red- and green-type Rubiscos.

## Red-type Rubiscos break the functional mould

Eukaryotic red-type Rubiscos tend to have high *S*_C/O_ with less of a trade-off for *k*_cat, C_ (Young *et al.*, 2016; Flamholz *et al.*, 2019). This is the most encouraging feature of red-type Rubisco kinetics—they break the canonical catalytic trade-offs reported for green-type Rubiscos, where an inverse relationship is observed between *S*_C/O_ and *k*_cat, C_ (Jordan and Ogren, 1983; Tcherkez *et al.*, 2006; Tcherkez, 2013), suggesting that red-type Rubisco kinetics may be unconstrained (or perhaps less constrained) than those of green-type Rubiscos. However, it is worth noting that recent studies probe more deeply into the correlations observed between Rubisco’s kinetic parameters. The high degree of sequence–structure relatedness between Rubiscos within the green lineage (Figs 1A, 2) means that kinetic measurements might not be considered to be independent, and thus any kinetic trade-offs observed for green-type Rubiscos may, at least in part, be a phylogenetic artefact (Bouvier *et al.*, 2021). However, analyses from Tcherkez and Farquhar (2021) could indicate that Rubisco kinetics are primarily driven by photosynthetic conditions. An additional study suggests that whilst the canonical *k*_cat, C_/*S*_C/O_ trade-off may be less strong than previously reported, both oxygenation and carboxylation *K*_cat_/*K*_M_ trade-offs remain, kinetic variability is highly limited, and Rubisco evolution remains mechanistically constrained (Flamholz *et al.*, 2019). We are excited for further study and discourse on this topic and, regardless, red-type Rubiscos remain kinetic outliers with impressively high specificity factors and decent catalytic turnover rates (Young *et al.*, 2016; Flamholz *et al.*, 2019).

Red-type Rubiscos also break the carbon isotope trends observed for green-type Rubiscos. Carbon fixation favours consuming ^12^C over the stable isotope ^13^C (von Caemmerer *et al.*, 2014), and is utilized as an indicator to identify autotrophic organisms and predict biochemical carbon fixation pathways employed by different organisms (Hanson *et al.*, 2014; Thomas *et al.*, 2019). Carbon isotope discrimination differences *in vivo* between organisms with red- and green-type Rubiscos reflect differences in their respective cellular environments, which may be CCM related (Wilkes and Pearson, 2019) or artefacts of culturing conditions (Brandenburg *et al.*, 2022). Carbon isotope discrimination in purified Rubisco is postulated to be a measure of the carboxylation transition state (carboxyketone) structure formed upon addition of CO_2_ to RuBP, with more and less product-like transition states forming in high *S*_C/O_ and high *k*_cat, C_ enzymes, respectively (Tcherkez *et al.*, 2006; Tommasi, 2021). Accordingly, Rubiscos with higher *S*_C/O_ display larger ^12^C/^13^C isotope effects, exhibiting a linear positive relationship (Tcherkez *et al.*, 2006). However, red-type Rubiscos break this green-type trend, with lower ^12^C/^13^C fractionation relative to the Form IA and IB green-type Rubiscos (Boller *et al.*, 2015; Thomas *et al.*, 2019). This suggests that the red-type and green-type Rubiscos may differentially stabilize Rubisco reaction intermediates (for a review, see Iñiguez *et al.*, 2020). However, these trends could reflect the phylogenetic constraints postulated by Bouvier *et al.* (2021). Further, carbon isotope discrimination values have only been reported for four red-type Rubiscos, and differences from study to study in the methodologies used for Rubisco kinetic measurements can weaken confidence in such trade-offs (Iñiguez *et al.*, 2021). Thus, these trends should be interpreted cautiously.

### Red-type Rubisco kinetics are desirable

The kinetic properties of red-type Form ID Rubiscos could provide opportunities to enhance photosynthetic carbon assimilation (PCA) in crop plants. With few exceptions, red-type Rubiscos exhibit much higher specificities than their green-type counterparts. Red algal Rubiscos exhibit specificity factors of 129–238, compared with 26–101 measured for all green lineage Rubiscos (Flamholz *et al.*, 2019; Table 1). Notably, CCM-less Form ID Rubiscos exhibit much higher specificity factors (excluding *Nannochloropsis* sp. Rubisco: 166.0–238.1), than eukaryotic Form IB green-type Rubiscos, regardless of presence (54.0–88.0) or absence (81.0–101.0) of a CCM (Table 1). A *k*_cat, C_ of 1.2–2.6 s^−1^ observed for red algal Rubisco falls within the 0.9–14.4 s^−1^ range observed for green lineage Rubiscos (0.9–6.7 s^−1^ for those not housed within a CCM). While there are limited kinetic data for Rubisco from brown algae, their *k*_cat, C_ and *K*_C_ values are comparable with those of red algae (Table 1; see Supplementary Table S1): Rubiscos from both red and brown algae have *K*_C_ values (3.3–23.6 µM) comparable with those exhibited by terrestrial plants (7.0–22.9 µM). Similarly, coccolithophorid Rubisco kinetics resemble those from diatoms. Wildly different kinetic properties were reported for the two microalgal ochrophytes *Nannochloropsis* sp. and *Olisthodiscus luteus*, which could represent the extensive diversity within this large phylum whose phylogeny is still under construction (see, for example, Barcytė *et al.*, 2021). Despite the high specificity values for certain red-type Rubiscos, it is carboxylation efficiency (*k*_cat, C_/*K*_C_^air^) improvements that are required to boost PCA in the context of the relatively low CO_2_ partial pressures in C_3_ chloroplasts (Whitney and Andrews, 2001; Andrews and Whitney, 2003). Excitingly, one red algal isoform, from *Griffithsia monilis*, has a superior carboxylation efficiency (206 s^−1^ mM^−1^) compared with Rubisco from C_3_ plants (122–138 s^−1^ mM^−1^). Modelling (Farquhar *et al.*, 1980) indicates that *G. monilis* Rubisco has the potential to boost PCA if transplanted into C_3_ chloroplasts by as much as 30% (Whitney *et al.*, 2001; Zhu *et al.*, 2004) (Table 1).

**Table 1.** Comparison of Rubisco kinetics from red- and green-type Rubisco lineages

Clade/phylum	Common names	Form	CCM	Organism	*k*_cat, C_	*K*_C_	S_C/O_	*k*_cat, O_/*K*_O_	*k*_cat, C_/*K*_C_^air^	Reference
					s^−1^	µM	–	s^−1^ mM^−1^	s^−1^ mM^−1^	
*Rhodophyta*	Redmacroalgae	ID	*–*	*Griffithsia monilis*	2.6	9.3	167.0	1.7	206	Whitney *et al.* (2001)
		ID	*–*	*Phycodrys rubens*	1.8	18.9				Iñiguez *et al.* (2019)
		ID	*–*	*Ptilota gunneri*	1.6	14.4				Iñiguez *et al.* (2019)
		ID	+	*Devaleraea ramentacea*	2.6	17.5				Iñiguez *et al.* (2019)
		ID	+	*Palmaria palmata*	2.1	15.9				Iñiguez *et al.* (2019)
		ID	+	*Palmaria decipiens*	2.4	17.4				Iñiguez *et al.* (2019)
	Red microalgae	ID	*–*	*Galdieria sulphuraria*	1.2	3.3	166.0	2.2	218	Whitney *et al.* (2001)
		ID	*–*	*Galdieria partitia*	1.6	6.6	238.1	1.0		Uemura *et al.* (1997)
		ID	*-*	*Cyanidium caldarium*	1.3	6.7	224.6	0.9		Uemura *et al.* (1997)
		ID	+	*Porphyridium purpureum*	1.4	22.0	143.5	0.5		Uemura *et al.* (1997)
		ID	+	*Porphyridium cruentum*	1.6	22.0	128.8	0.6		Read and Tabita (1994)
*Ochrophyta*	Brownmacroalgae	ID	+	*Alaria esculenta*	2.1	23.6				Iñiguez *et al.* (2019)
		ID	+	*Desmarestia aculeata*	1.4	13.3				Iñiguez *et al.* (2019)
		ID	+	*Laminaria solidungula*	1.6	18.5				Iñiguez *et al.* (2019)
		ID	+	*Laminaria digitata*	1.4	17.0				Iñiguez *et al.* (2019)
		ID	+	*Saccharina latissima*	1.8	19.4				Iñiguez *et al.* (2019)
		ID	+	*Himantothallus grandifolius*	2.1	18.1				Iñiguez *et al.* (2019)
	–	ID	+	*Nannochloropsis* sp.	1.0*	7.0	27.0	4.6		Tchernov *et al.* (2008)
	–	ID	?	*Olisthodiscus luteus*	0.8	59.0	100.5	0.2		Read and Tabita (1994)
	Diatom	ID	*+*	*Cylindrotheca* N1	0.8	31.0	105.6	0.3		Read and Tabita (1994)
		ID	*+*	*Cylindrotheca fusiformis*	2.0	36.0	110.8	0.4		Read and Tabita (1994)
		ID	*+*	*Phaeodactylum tricornutum*	3.4	27.9	113.0	1.1		Whitney *et al.* (2001)
		ID	*+*	*Thalassiosira weissflogii* CCMP 1336	3.2	65.0	79.0	0.6	44	Young *et al.* (2016)
		ID	*+*	*Thalassiosira oceania* CS-427	2.4	65.0	80.0	0.4	29	Young *et al.* (2016)
		ID	*+*	*Skeletonema marinoi* CCMP 1332	3.2	68.0			36	Young *et al.* (2016)
		ID	*+*	*Chaetoceros calcitrans* CCMP 1315	2.6	25.0	57.0	1.9	63	Young *et al.* (2016)
		ID	*+*	*Chaetoceros muelleri* CCMP 1316	2.4	23.0	96.0	1.2	65	Young *et al.* (2016)
		ID	*+*	*Chaetoceros calcitrans* CS-178	3.4	31.0	75.0	1.4	72	Young *et al.* (2016)
		ID	*+*	*Bellerochea* cf*. horologicalis* CS-874/01	2.1	50.0			31	Young *et al.* (2016)
		ID	*+*	*Phaeodactylum tricornutum* UTEX 642	3.2	36.0	108.0	0.8	62	Young *et al.* (2016)
		ID	*+*	*Phaeodactylum tricornutum* CS-29	3.3	41.0	116.0	0.8	58	Young *et al.* (2016)
		ID	*+*	*Fragilariopsis cylindrus* CCMP 1102	3.5	64.0	77.0	0.7	40	Young *et al.* (2016)
		ID	*+*	*Cylindrotheca fusiformis* CS-13	3.7		79.0			Young *et al.* (2016)
		ID	*+*	*Thalassiosira hyalina*	4.1*	50.0	99.0	0.9		Valegård *et al.* (2018)
		ID	*+*	*Bacterosira bathyomphala*	4.6*	81.0	87.0	0.7		Valegård *et al.* (2018)
		ID	*+*	*Skeletonema marinoi*	4.6*	48.0	96.0	1.0		Valegård *et al.* (2018)
		ID	*+*	*Thalassiosira nordenskioeldii*	4.7*	122.0	82.0	0.5		Valegård *et al.* (2018)
		ID	*+*	*Thalassiosira antarctica*	3.7*	93.0	90.0	0.5		Valegård *et al.* (2018)
		ID	*+*	*Fragilariopsis cylindrus*	0.39 (3 °C)	50.0				Young *et al.* (2015)
*Haptista/Haptophyta*	Coccolithophorid	ID	+	*Pleurochrysis carterae*	3.3	17.7	102.0	1.9	108	Heureux *et al.* (2017)
	–	ID	+	*Tisochrysis lutea*	2.2	24.1	89.0	1.0	68	Heureux *et al.* (2017)
	–	ID	?	*Pavlova lutheri*	2.5	14.5	125.0	1.4	140	Heureux *et al.* (2017)
*Proteobacteria*	Alpha-proteobacteria	IC	–	*Rhodobacter sphaeroides*	3.7	59.7	58.4	0.8	54	Gunn *et al.* (2020)
	Beta-proteobacteria	IC	–	*Cupriavidus necator*	2.1	50.2	74.0	0.6		Lee *et al.* (1991)
	Nitrogen fixing	IC	–	*Bradyrhizobium japonicum*	2.2	50.2	74.8	0.6		Horken and Tabita (1999a)
		IC	–	*Xanthobacter flavus*	1.4	76.1	44.4	0.4		Horken and Tabita (1999a)
*Cyanobacteria*	Cyanobacteria	IB	+	*Synechococcus elongatus* PCC 6301	9.8	152.0	50.3	1.3	53	Shih *et al.* (2016)
		IB	+	*Synechococcus sp.* PCC 7002	8.6*	119.0	43.3	1.7		Ninomiya *et al.* (2008)
		IB	+	*Synechocystis* PCC 6803	14.3				53	Marcus *et al.* (2011)
		IA	+	*Prochlorococcus marinus* MIT 9313	6.6	309.0	59.9	0.6	18	Shih *et al.* (2016)
*Proteobacteria*	–	IA	?	*Allochromatium vinosum*	6.7	37.0	41.0	4.4		Jordan and Chollet (1985)
	–	IA	+	*Hydrogenovibrio marinus* (carboxysome operon *CbbL2S2*)	2.0		38.4			Hayashi *et al.* (1998)
	–	IA	–	*Hydrogenovibrio marinus* (operon *CbbL1S1*)	0.9		30.9			Hayashi *et al.* (1998)
	–	IA	–	*Rhodobacter capsulatus*	2.5	22.1	25.9	4.5		Horken and Tabita (1999b)
	–	IA	–	*Thiobacillus denitrificans*	1.4	105.0	53.4	0.2		Hernandez *et al.* (1996)
*Streptophyta* C_3_ plants	Tobacco	IB	–	*Nicotiana tabacum*	3.1	9.7	82.0	3.9	138	Whitney *et al.* (2015)
	Arabidopsis	IB	–	*Arabidopsis thaliana*	3.0	9.8	80.0	3.8	125	Whitney *et al.* (2015)
	–	IB	–	*Flaveria pringlei*	3.5	13.7	81.0	2.7		Whitney *et al.* (2011)
	Wheat	IB	–	*Triticum aestivum*	3.0	10.9	100.0	2.6		Carmo-Silva *et al.* (2010)
	Rice	IB	–	*Oryza sativa* ssp. *Indica*	2.2	7.0	101.0	2.6	122	Orr *et al.* (2016)
*Streptophyta* C_4_ plants	–	IB	+	*Flaveria bidentis*	4.8	20.4	81.0	2.9		Whitney *et al.* (2011)
	Sorghum	IB	+	*Sorghum bicolor*	5.8	22.9			175	Sharwood *et al.* (2016a)
	Maize	IB	+	*Zea mays*	5.5	18.9	88.0	2.0	177	Sharwood *et al.* (2016a)
	Lawngrass	IB	+	*Zoysia japonica*	4.4	18.5	84.1	2.8		Carmo-Silva *et al.* (2010)
	Grass	IB	+	*Setaria viridis*	5.9	18.1	72.7	4.4	231	Sharwood *et al.* (2016b)
*Chlorophyta*	Green algae	IB	+	*Chlamydomonas reinhardtii*	1.8*	30.0	64.0	1.0		Zhu and Spreitzer (1994)
		IB	+	*Chlamydomonas reinhardtii*	2.3*	35.0	63.0	1.0		Spreitzer *et al.* (2005)
		IB	+	*Scenedesmus obliquus*		38.0	63.0			Savir *et al.* (2010)
		IB	–	*Coccomyxa*sp.		11.9	82.9			Palmqvist *et al.* (1995)
*Discoba*		IB	+	*Euglena gracilis*		25.0	54.0			Savir *et al.* (2010)

Measurements of catalytic constants for substrate-saturated rates of carboxylation (*k*_cat, C_), specificity for CO_2_ over O_2_ (*S*_C/O_) [i.e. (*k*_cat, C_**×***K*_O_**)/**(*k*_cat, O_**×***K*_C_)], carboxylation efficiency (*k*_cat, C_/*K*_C_^air^), and oxygenation efficiency *k*_cat, O_/*K*_O_ were collected or calculated from published data. Values of *k*_cat, C_ calculated using the molecular weight of the specific Rubisco, estimated from LSu and SSu UniProt sequences, are denoted by an asterisk. All available kinetic values are included from red-type Rubiscos. Selected representatives from green-type Rubisco lineages are included for comparison. See Supplementary Table S1 for references and the full table including available kinetic measurements for *k*_cat, C_/*K*_C_, Michaelis–Menten constants for CO_2_ (*K*_C_) and O_2_ (*K*_O_), and CO_2_ under atmospheric oxygen (*K*_C_^air^), and substrate-saturated rates of oxygenation (*k*_cat, O_).

### Do red-type Rubiscos exhibit reduced oxygen sensitivity?

Red-type Rubiscos might exhibit somewhat reduced O_2_ sensitivity compared with green-type Rubiscos. Eukaryotic red-type Rubiscos (Form ID) exhibit higher *K*_O_ values (360–2000 µM), and thus tend to have lower affinity for oxygen than eukaryotic green-type Rubiscos (170–660 µM; Table 1). No clear trend exists for *k*_cat, O_ between the red- and green-type Rubiscos. In general, this parameter is under-reported and often calculated from other kinetic parameters rather than being directly measured. However, red-type Rubiscos tend to exhibit lower oxygenation efficiencies (*k*_cat, O_/*K*_O_) (Table 1; see Supplementary Table S1). Excluding the suspiciously high value reported for *Nannochloropsis* sp. Rubisco, all plastid-evolved red-type Rubiscos have oxygenation efficiencies of 0.2–2.2 s^−1^ mM^−1^, while Rubiscos from C_3_ and C_4_ plants exhibit *k*_cat, O_/*K*_O_ values between 2.0 s^−1^ mM^−1^ and 3.9 s^−1^ mM^−1^. This lower red-type Rubisco oxygen sensitivity extends to Form IC Rubiscos with oxygenation efficiencies in the range of 0.4–0.8 s^−1^ mM^−1^. At the whole-cell level, non-green algae have lower rates of light-dependent O_2_ consumption than green algae and C_3_ plants under both CO_2_-limiting and saturating conditions (Badger *et al.*, 1998). While Rubisco is not the only factor contributing to light-dependent O_2_ evolution (e.g. photoreduction), these observations are consistent with the lower oxygen sensitivity measured for red-type Rubiscos (Table 1). It is proposed that the βE–βF SSu hairpins could reduce oxygenation transition state stability or increase the activation energy for the Rubisco oxygenation reaction (Shibata *et al.*, 1996; Uemura *et al.*, 1997). Form IC Rubiscos exhibit higher *S*_C/O_ values than Form IA and IB cyanobacterial Rubiscos (Iñiguez *et al.*, 2020), and higher specificity than and comparable catalytic turnover rates with Form IA proteobacterial Rubisco (Table 1). All Form IC kinetics fall within the range of measured values for diatoms. These trends could support the idea that the ancestral red-type Rubisco exhibited high specificity for CO_2_, compared with the green-type Rubisco progenitor. However, prokaryotic green-type Rubiscos tend to exhibit higher values for *K*_O_ and lower oxygenation efficiencies than eukaryotic green-type Rubiscos, and thus it could be that these oxygenation kinetic differences reflect CCM efficiency.

### Red-type Rubisco adaption or maladaption to environmental conditions

It has been proposed that all Rubiscos have optimized their kinetic properties to adapt to their gaseous environment (Tcherkez *et al.*, 2006). For example, Rubiscos in environments enriched in CO_2_ tend to have higher *k*_cat, C_ offset by lower *S*_C/O_ and CO_2_ affinity (i.e. a higher *K*_C_), which is especially apparent when comparing C_3_ and C_4_ species (Christin *et al.*, 2008) (Table 1). These offsets are possible, without detriment to organism PCA and growth, because of relaxed evolutionary constraints on *S*_C/O_ and *K*_C_ as a consequence of the CCM strategies employed by these organisms (Price *et al.*, 2008).

Non-green algae from hot environments (i.e. thermal springs) have higher *S*_C/O_ values than red algae from more temperate environments, which is advantageous for carbon fixation as the relative solubility of CO_2_ decreases compared with O_2_ with increasing temperatures (Smith, 1928) (Table 1). Diatom Rubiscos have also clearly adapted to their pyrenoid environment, with a reduction in *S*_C/O_ and increase in *k*_cat, C_ compared with eukaryotic red-type Rubiscos that lack any form of CCM. However, their Rubiscos do not show a positive relationship between *k*_cat, C_ and *K*_C_, which again highlights that red-type Rubiscos do not conform to the kinetic rules written by green-type Rubiscos. Overall, the vast majority of assayed diatom Rubiscos exhibit *S*_C/O_ values higher than cyanobacteria and C_4_ species. Most diatom Rubiscos also retain higher CO_2_ specificity than Rubiscos found in the CCM-lacking C_3_ chloroplast.

A striking adaptation outlier is the anoxygenic phototroph *Rhodobacter sphaeroides* (Imhoff *et al.*, 2018) that expresses virtually no Rubisco under aerobic conditions (Zhu and Kaplan, 1985; Jouanneau and Tabita, 1986). Despite operating under anaerobic conditions, *R. sphaeroides* Rubisco exhibits low oxygenation efficiency (Table 1). However, interpretation of the evolutionary implications of *R. sphaeroides* Rubisco’s low sensitivity to oxygen is limited by data availability, which could be resolved by a more extensive catalytic survey of proteobacterial red-type Rubiscos. Complete and wide kinetic analyses are important to observe and interpret key kinetic trends across and within lineages, and how these functional trends might relate to Rubisco sequence–structure. Differences in assay conditions between different studies also pose a significant challenge to the ability to draw meaningful conclusions about Rubisco structure–function trends (see Iñiguez *et al.*, 2021).

## Red-type Rubisco biogenesis

### Transcription

Transcription of the *cbb* operon in proteobacteria (Form IA and Form IC) is primarily controlled by the LysR-type transcriptional regulator (for a review, see Maddocks and Oyston, 2008), CbbR. In cyanobacteria, the Rubisco operon and carboxysomal genes (Price *et al.*, 2008) are regulated by RbcR. The eukaryotic RbcR homologue, called Ycf30, is encoded in the plastome of organisms with Form ID Rubisco (Minoda *et al.*, 2010). *CbbR*, *RbcR*, and *Ycf30* are generally located upstream of the *rbcLS* operon (Fig. 1C). Notably, *Ycf30* is of cyanobacterial origin and not a remnant of primary or secondary endosymbiotic events (Maier *et al.*, 2000). While there is variability in the specific metabolite effectors for CbbR, RbcR, and Ycf30 from different organisms, they are very generally controlled by light and CO_2_ concentration (van Keulen *et al.*, 1998; Grzeszik *et al.*, 2000; Dubbs *et al.*, 2004; Nishimura *et al.*, 2008; Minoda *et al.*, 2010). Cognate transcriptional regulators do not limit eukaryotic (Whitney *et al.*, 2001; Lin and Hanson, 2018) or proteobacterial (Joshi *et al.*, 2015; Gunn *et al.*, 2020) red-type Rubisco in heterologous systems.

However, a deeper appreciation of the green transcriptional regulatory system (Atkinson *et al.*, 2017; Khumsupan *et al.*, 2020) might be more appropriate for engineering approaches, allowing us to hack the existing regulatory systems relevant to the CO_2_-fixing needs of the host system.

### Unique post-translational modifications

Post-translational modifications (PTMs) can influence Rubisco stability, structure, and activity (Apel *et al.*, 2010), a number of which have been identified from interpretation of electron density in crystallographic data (Table 2; Fig. 2A). Valegård *et al.* (2018) published the first four diatom Rubisco structures revealing extensive LSu PTMs compared with green-type Rubiscos. Hydroxylation of buried residues (48, 155, 174, and 198, numbered relative to the *Thalassiosira antarctica* sequence, Fig. 2A), including N-terminal domain residues at the dimer–dimer interface (109 and 150) probably contribute to holoenzyme stability. Residue 155 is also hydroxylated in *Chlamydomonas reinhardtii*, but no hydroxylation modifications are observed in any other green-type Rubisco structure. Solvent-exposed PTMs in diatom structures include a trimethylated Lys346 close to loop 6 and a nitrosylated Cys457. Nitrosylated cysteines are also observed in *Galdieria sulphuraria* Rubisco at residues 176 and 457. Cysteine is highly conserved in red-type Rubiscos at residue 457, and in all green lineage Rubiscos at position 176. Nitrosylation can attenuate Rubisco activity in red algae and higher plants (Abat and Deswal, 2009; Stec, 2012) (Fig. 2A), and hints at the involvement of nitric oxide signalling in redox regulation of red-type Rubisco. While these cysteine PTMs are not observed in the green lineage, disulfide bonds between highly conserved cysteine residues, including residue 176, protect Rubisco from oxidative and/or salt stress in land plants and green algae (Mehta *et al.*, 1992; Marcus *et al.*, 2003; Li *et al.*, 2004; Moreno *et al.*, 2008). Disulfide bonds in the green lineage and nitrosylation in the red lineage at equivalent LSu cysteine positions suggest that the red lineage may similarly use these cysteines (albeit through a different mechanism) to protect against stress and/or regulate Rubisco activity. Differences in the occupancy (or indeed absence) of PTMs at equivalent amino acid residues between the diatom and non-green algal Rubisco structures could perhaps be explained by cautious interpretation of lower resolution structural data, represent divergence between species, or reflect variation in differences in the environmental conditions in which the diatoms were harvested. In a similar vein, all available diatom Rubisco structures are derived from Arctic species, and thus analyses of diatoms from more diverse environments are required to determine if these PTMs are broadly observed across all diatom species.

**Table 2.** Rubisco post-translational modifications

Lineage		Rubisco form	Species	PDB code(s)	Resolution(s) (Å)	LSu PTMs—residue number
Red-type	Red algae	ID	*Galdiera partitia*	1BWV, 1IWA	2.40, 2.60	nd.
		ID	*Galdieria sulphuraria*	4F0H, 4F0M, 4F0K	1.96, 2.25, 2.05	CYS to SNC—181 (176) CYS to SNC—460 (457)
	Diatom	ID	*Chaetoceros socialis*	5OYA	1.80	PRO to HYP—48 (48) CYS to CSO—109 (109) LYS to LOH—150 (150) PRO to HYP—155 (155) LEU to HL2— 174 (174) LYS to M3L—346 (346) CYS to SNC—457 (457)
		ID	*Skeletonema marinoi*	6FTL	2.60	Not modelled—109 (109) LYS to LOH—150 (150) PRO to HYP—155 (155) LEU to HLU—174 (174) LYS to LYO—198 (198) LYS to M3L—346 (346) Not modelled—457 (457)
		ID	*Thalassiosira antarctica* var*. borealis*	5MZ2	1.90	PRO to HYP—48 (48) CYS to CSO—109 (109) LYS to LYO—150 (150) PRO to HYP—155 (155) LEU to HLU—174 (174) LYS to LYO—198 (198) LYS to M3L— 346 (346) Not modelled—457 (457)
		ID	*Thalassiosira hyalina*	5N9Z	1.90	PRO to HYP—48 (48) CYS to CSO—109 (109) LYS to 8RE—150 (150) PRO to HYP—155 (155) LEU to HLU—174 (174) LYS to LYO—198 (198) LYS to M3L—346 (346) Not modelled—457 (457)
	Proteobacteria	IC	*Cupriavidus necator*	1BXN	2.70	nd
		IC	*Rhodobacter sphaeroides*	5NV3	3.39	nd
Green-type	Vascular plant	IB	*Arabidopsis thaliana*	5IU0	1.50	nd
		IB	*Nicotiana tabacum*	1EJ7, 3RUB, 1RLD, 1RLC, 4RUB	2.45, 2.00, 2.50, 2.70, 2.70	nd
		IB	*Oryza sativa*	3AXM, 6KYI, 1WDD, 3AXK	1.65, 1.75, 1.35, 1.90	nd
		IB	*Pisum sativum*	4HHH, 4MKV	2.20, 2.15	nd
		IB	*Spinacia oleracea*	8RUC, 1IR1, 1UPP, 1UPM, 1AA1, 1RXO, 1RCX, 1RCO, 1RBO, 1AUS	1.60, 1.80, 2.30, 2.30, 2.20, 2.20, 2.40, 2.30, 2.30, 2.20	nd
		IB	*Triticum aestivum*	5WSK	1.78	nd
	Green algae	IB	*Chlamydomonas reinhardtii*	1GK8, 7JN4, 1IR2	1.40, 2.68, 1.84	PRO to HYP—104 (108) PRO to HYP—151 (155) CYS to SMC—256 (260) CYS to SMC—369 (372)
	Cyanobacteria	IB	*Synechococcus elongatus* PCC 6301	1RSC, 1RBL	2.30, 2.20	nd
		IB	*Thermosynechococcus elongatus* BP-1	2YBV, 3ZXW	2.30, 2.10	nd

Post-translational modifications (PTMs) of large subunit residues identified in Form I red and green lineage Rubiscos from X-ray crystallographic and CryoEM data deposited in the Protein Data Bank (PDB). The nature and position of PTMs are indicated, with residue numbering in parentheses indicating the equivalent residue numbering in *Thalassiosira antarctica* Rubisco (PDB: 5MZ2, also see Fig. 2A). PTM abbreviations: SNC, *S*-nitroso-cysteine; HYP, 4-hydroxyproline; CSO, *S*-hydroxycysteine; LOH, 3,4-dihydroxylysine; HL2, (2*S*,3*R*)-2-amino-3-hydroxy-4-methylpentanoic acid; M3L, *N*-trimethyllysine; HLU, beta-hydroxyleucine; LYO, 4-hydroxylysine; 8RE, 3,4-hydroxylysine; SMC, *S*-methylcysteine; nd, not detected. The carbamylated catalytic lysine present in activated Rubisco across all lineages (lysine carboxylic acid; KCX) is intentionally excluded

PTMs located on N-terminal LSu residues are usually not observed by structural methods, because the first residues are often missing from Rubisco LSu density, and these have thus far been identified using analytical approaches. N-terminal PTMs are highly conserved in chloroplast Rubiscos where they are, more specifically, co-translational modifications (for a review, see Houtz *et al.*, 2008). These PTMs include deformylation of Met1, peptidase removal of Met1 and Ser2, acetylation of Pro3, and often trimethylation of Lys14 (tobacco Rubisco numbering), and may protect Rubisco from proteolysis (Apel *et al.*, 2010). Additional N-terminal PTMs might be present in red-type Rubiscos that have not yet been detected. Indeed N-terminal blocking of Edman sequencing of *P. tricornutum* and *G. sulphuraria* Rubisco LSus (Whitney *et al.*, 2001) suggests that this might be the case.

### Folding and assembly

The LSu interacts with a series of chaperones, both during and after translation, within the plastid stroma. These include homologues of Hsp70, DnaJ, and GrpE (Goloubinoff *et al.*, 1989; Liu *et al.*, 2010; Hartl *et al.*, 2011), which notably do not limit the assembly of green-type Rubisco in *Escherichia coli* (Aigner *et al.*, 2017; Lin *et al.*, 2020). LSus subsequently associate with chaperonin folding cages: the GroEL/GroES chaperonin complex in prokaryotes, and the Cpn60/Cpn10 or Cpn60/Cpn20 complex in eukaryotes (Hartl *et al.*, 2011). Eukaryotic green-type Rubiscos additionally require a suite of assembly factors—Raf1, Raf2, RbcX, and BDS2 that stabilize LSu intermediates before SSu binding to the L_8_ core (Liu *et al.*, 2010; Feiz *et al.*, 2012; Aigner *et al.*, 2017). Homologues of these chaperones are not found in organisms expressing red-type Rubiscos. The ability of red-type Rubisco βE–βF hairpins to supplant the function of RbcX has been established using hairpin sequences from *R. sphaeroides* Rubisco (Form IC), which can assemble in *E. coli* and tobacco without the need for additional chaperones (Joshi *et al.*, 2015; Gunn *et al.*, 2020). However, attempts at heterologous expression of Form ID Rubiscos have thus far failed, indicating that additional chaperones are required for assembly, and are a major factor limiting the functional expression of eukaryotic red-type Rubiscos in chloroplasts (Whitney *et al.*, 2001; Lin and Hanson, 2018).

## Red-type Rubisco activation

### CbbX keeps Red-type Rubisco active

Rubisco activity is regulated by nuclear-encoded metabolic repair proteins, called Rubisco activase (RCA) in higher plants and β-cyanobacteria, that keep Rubisco in its active state by removing inhibitory sugar phosphates that can bind the Rubisco active site (for a review, see Bhat *et al.*, 2017). Organisms with a red-type Rubisco have a similar, but distinct, Rubisco activase protein called CbbX. RCA and CbbX are both members of the AAA+ protein family and thus require ATP for activity (for a review, see Houtz and Portis, 2003). A number of protein structures of RCA, CbbX, and Rubisco–RCA complexes have contributed to our understanding of activase function (Henderson *et al.*, 2011; Mueller-Cajar *et al.*, 2011; Stotz *et al.*, 2011; Hasse *et al.*, 2015; Flecken *et al.*, 2020; Tsai *et al.*, 2020). Functional RCA and CbbX both adopt a hexameric ring structure (Blayney *et al.*, 2011; Mueller-Cajar *et al.*, 2011; Stotz *et al.*, 2011). However, RCA and CbbX often adopt an oligomeric helical conformation in crystal structures, and in solution, which may represent a storage form (Mueller-Cajar *et al.*, 2011; Serban *et al.*, 2018). Unlike RCA, prokaryotic CbbX function is under allosteric control by RuBP (Stotz *et al.*, 2011). In eukaryotic CbbX, RuBP enhances ATP hydrolysis, rather than providing allosteric control (Loganathan *et al.*, 2016). In the presence of ATP, RCA exists as a hexamer (Keown and Pearce, 2014), whereas prokaryotic CbbX requires both ATP and RuBP to adopt this functional conformation (Mueller-Cajar *et al.*, 2011).

Rubisco activases interact with the Rubisco LSu by threading terminal LSu residues through the pore of hexameric RCA or CbbX. By tugging on the LSu, these activases interfere with the conformation of the inhibited Rubisco complex, allowing the release of inhibitors from the active site (for a review, see Bhat *et al.*, 2017). While both RCA and CbbX perform the same function, the mechanism and interactions with their respective Rubiscos are distinct. CbbX interacts with a conserved flexible C-tail extension in red-type Rubisco LSus to invoke inhibitor release (Mueller-Cajar *et al.*, 2011; Loganathan *et al.*, 2016) (Fig. 2A). In contrast RCA interacts with the conserved green-type Rubisco N-terminal LSu residues, resulting in a cascade effect that disrupts loop 6 closed over the inhibitory sugar in the active site (Flecken *et al.*, 2020; Ng *et al.*, 2020).

While CbbXs in prokaryotic and eukaryotic red-type Rubisco-containing species are related, CbbX is also widely distributed across α-cyanobacterial species and found in tandem with RCA in certain β-cyanobacteria (Zarzycki *et al.*, 2013) (Fig. 1C). It is likely that eukaryotic CbbX is of proteobacterial origin and was transferred to red lineage plastids concomitantly with the horizontal gene transfer of *cbb*LS (Maier *et al.*, 2000). In red algae and cryptophytes, *CbbX* is located downstream of Rubisco-encoding genes, in the *rbcLS* operon (Reith and Munholland, 1995; Ohta *et al.*, 1997; Douglas and Penny, 1999), while in heterokonts and diatoms, *CbbX* is located distantly from the Rubisco-encoding genes in the plastid (Kowallik *et al.*, 2007). Higher plants code for two RCA isoforms, which exhibit distinct ATP and temperature responses, and can form heterooligomers (for a review, see Carmo-Silva *et al.*, 2015). Red lineage eukaryotes have similarly undergone a gene duplication event, resulting in both a nuclear and a plastid copy, both of which may be necessary for maximal activation (Maier *et al.*, 2000). Overexpressing nuclear-encoded CbbX boosts photosynthesis in the non-green algal species *Nannochloropsis oceanica* (Wei *et al.*, 2017). In *Cyanidioschyzon merolae* (a red alga), both plastid and nuclear CbbX copies are required for functionality, forming a heterooligomeric complex in 1:1 stoichiometry (Loganathan *et al.*, 2016).

### CbbX activity in chloroplasts

A larger percentage of the total *R. sphaeroides* Form IC Rubisco pool is activated (i.e. has no inhibitory ligand bound at the active site) under elevated CO_2_ conditions in chloroplasts (Gunn *et al.*, 2020). This trend is observed regardless of the presence or absence of its cognate CbbX, albeit with higher activation in the presence of CbbX. This is in stark contrast to higher plant Rubiscos whose activation status decreases with increasing CO_2_, which may be a response to a reduction in electron transport products and/or related changes in pH across the thylakoid membrane (Whitney *et al.*, 1999). These opposing trends could represent differences in ATPase capacity between RCA and CbbX. However, because this trend is observed in both the presence and absence of CbbX, and because red-type Rubisco exhibits different rates of inhibitor binding and release (Pearce, 2006), we speculate that this could represent a difference in the capacity of the red-type Rubisco active site to bind inhibitory sugars under different CO_2_ pressures. What is clear is that there is a requirement to provide red-type Rubisco with a compatible CbbX for maximal activation of introduced red-type Rubiscos in heterologous systems, and—for Form ID Rubiscos specifically—both nuclear and plastid copies may be necessary (Gunn *et al.*, 2020).

## Using red-type Rubiscos to enhance crop yield: progress, opportunities, and challenges

**Fig. 4.**
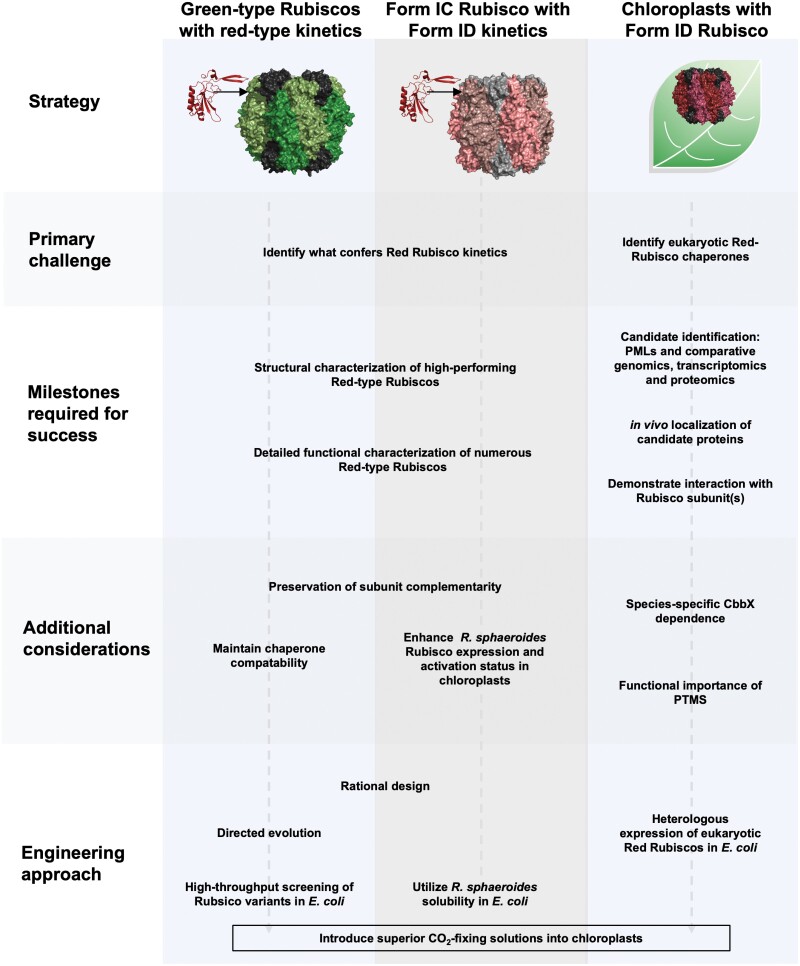
Schematic of possible routes to engineer red-type Rubisco kinetics into green plants. Red-type Rubisco kinetics could be introduced into green plants by introducing ‘red’-like sequence structure into (i) green-type Rubiscos or (ii) the chloroplast-competent proteobacterial Rubisco from *R. sphaeroides*, or by (iii) identifying the full complement of accessory proteins required to functionally express a high-performing eukaryotic red-type Rubisco in chloroplasts. Experimental challenges and engineering approaches for each of these strategies are indicated. Abbreviations: PML, photosynthetic mutant library.

Utilizing certain red-type Rubisco structure–function has exciting potential to boost PCA and thus crop yield in green plants. We suggest three routes to conferring ‘redness’ to green chloroplasts: (i) modify green-type Rubisco to exhibit kinetic characteristics of red-type Rubiscos; (ii) transplant a more ‘primitive’ red-type Rubisco isoform into chloroplasts and engineer this isoform towards more eukaryotic red-type kinetic properties; or (iii) transplant a functional high performing eukaryotic red-type Rubisco variant into chloroplasts. The progress and challenges for each of these strategies are discussed below, and summarized in Fig. 4.

### Engineer green-type Rubiscos to be more like red-type Rubiscos

A greater number of red-type Rubisco sequences, structural models, and kinetic data to pinpoint catalysis-enhancing sequence–structure could provide a route to rationally engineer green-type Rubiscos to imitate red-type Rubisco kinetic properties, while retaining their interactions with their cognate chaperones and thus their chloroplast solubility. There has already been moderate success transplanting red algal sequence into green algal Rubisco for enhanced catalytic performance (Read and Tabita, 1994). Initial engineering approaches could focus on sequence–structural variation in regions known to influence catalysis, such as the LSu loop 6, C-terminal residues, and/or the red latch residue. The kinetic impairment upon transplanting the SSu βE–βF hairpin into other Rubiscos suggests that complementary changes are required elsewhere in the holoenzyme to functionally accommodate this structure (Spreitzer *et al.*, 2005; Joshi *et al.*, 2015). Huge leaps have been made in recent years in our understanding of green-type Rubisco chaperone requirements (for a review, see Wilson and Hayer-Hartl, 2018), and could be used to map the sequence space in which modifications must be avoided to maintain their chaperone interactions and provide some initial engineering constraints. Making green-type Rubisco kinetics mirror those of red-type Rubiscos is made more feasible with the advent of the two synthetic biology expression systems for higher plant Rubisco in *E. coli* (Aigner *et al.*, 2017; Lin *et al.*, 2020), which greatly increase the throughput of Rubisco manipulation.

### Enhance kinetic properties of chloroplast-soluble *R. sphaeroides* Rubisco

Unlike red algal and diatom Rubiscos which fail to assemble in chloroplasts, the proteobacterial red-type Rubisco from *R. sphaeroides* assembles readily in both *E. coli* and chloroplasts (Gunn *et al.*, 2020). While *R. sphaeroides* Rubisco kinetic properties are insufficient to enhance PCA in chloroplasts, it can be utilized as a chloroplast-soluble red-type Rubisco scaffold that can be engineered towards higher carboxylation efficiency by augmenting its sequence–structure with that from Form ID Rubiscos. There are already viable routes to improving *R. sphaeroides* Rubisco kinetics. Hybrid Rubiscos containing *R. sphaeroides* LSus and SSus from eukaryotic red-type Rubiscos exhibit dramatically altered kinetics (Joshi *et al.*, 2015; Gunn *et al.*, 2020). This suggests that more targeted SSu changes (i.e. rational design) could yield improved kinetics. The ability to test the folding/assembly of *R. sphaeroides* Rubisco in *E. coli* (Gunn *et al.*, 2020) benefits directed evolution approaches, which have had initial success producing *R. sphaeroides* Rubisco with 11% and 27% increases in carboxylation efficiency and carboxylation rate, respectively (Zhou and Whitney, 2019). In addition to catalytic improvements, there is room to optimize expression and activity of *R. sphaeroides* Rubisco in chloroplasts. *Rhodobacter sphaeroides* Rubisco expression levels in chloroplasts are lower than that of tobacco Rubisco (Gunn *et al.*, 2020), and exploiting a stronger promoter or introducing additional gene copies could boost expression. Moreover, the lower carbamylation status of *R. sphaeroides* compared with tobacco Rubisco in chloroplasts could represent a limitation to CbbX availability, which could be circumvented by overexpression (Wei *et al.*, 2017). A lower activation status could also be indicative of suboptimal CbbX modulation in the chloroplast because of differences in the availability of ATP and/or RuBP compared with the *R. sphaeroides* cytosol, or reflect some other fundamental distinction between the activation mechanism of red and green lineage Rubiscos.

### Transplant a high performing red algal Rubisco into a green plant

Nature has already evolved at least one red-type Rubisco isoform that could enhance PCA in chloroplasts: Rubisco from *G. monilis*. See Sharwood (2017) for an elegant illustration of the photosynthetic carbon assimilation advantage expected from expressing *G. monilis* Rubisco in either C_3_ chloroplasts or C_4_ bundle sheath cells. However, two key studies indicate that *G. monilis*, *G. sulphuraria*, and *Phaeodactylum tricornutum* Rubisco do not assemble in tobacco chloroplasts (Whitney *et al.*, 2001; Lin and Hanson, 2018). These red-type Rubiscos accumulate in high abundance (5–30% of leaf protein) in insoluble fractions, and within the chloroplast. It has been suggested that assembly could have been impeded by Rubisco subunit interactions with extant green-type chaperones or other plastome-located proteins (Joshi *et al.*, 2015), or because of a strict requirement for cognate (or suite of) red-type Rubisco chaperone(s) (Whitney *et al.*, 2001; Lin and Hanson, 2018). It is likely that the latter is true as neither study detected higher molecular weight complexes indicative of incompatible binding of (green-type) chaperonins and chaperones to red-type Rubisco subunits.

In order to successfully transplant a functional eukaryotic red-type Rubisco into chloroplasts, we first need to understand the chaperone requirements for red-type Rubiscos. While red plastid genomes encode a Cpn60 chaperonin isoform, and a DnaK (Hsp70) chaperone (Reith and Munholland, 1995), it is not known if these are sufficient to fold rhodophyte Rubisco in heterologous systems. Further, given the divergence in plastome environment, red-type Rubiscos already having an in-built RbcX (βE–βF hairpin), and the time frame in which the organisms evolved since the divergence of the red and green plastid lineages, it is perhaps reasonable to speculate that rhodophytes have evolved a set of chaperones that have no homologues to those found in chlorophytes. The possibility also exists that there may be plastid lineage- and/or species-specific chaperone requirements. For example, individual Arabidopsis chaperonins/chaperones show variability in their ability to substitute for those from tobacco (Lin *et al.*, 2020).

Many of the green-type chaperones were identified via maize photosynthetic mutant libraries (for a review, see Wilson and Hayer-Hartl, 2018), and a large mutant library for green algae has been used to identify previously uncharacterized genes involved in photosynthesis (Li *et al.*, 2019). A similar approach could be fruitful if applied to red algae. In addition to harnessing the power of comparative analysis of the growing number of available rhodophyte genomes (Blaby-Haas and Merchant, 2019), proteomic approaches could identify candidate chaperone proteins present in the rhodoplast stroma that could thus interact with Rubisco during biogenesis. Transcriptome data indicate that red algal Rubisco expression is light induced (Minoda *et al.*, 2010), and further analyses of algal tissue harvested under different growth conditions and/or developmental stages could be informative. Biochemical approaches could be employed to capture intermediate Rubisco–chaperone complexes. Co-localization studies to verify overlapping subcellular location with Rubisco would be a useful first-pass functional evaluation for putative chaperones—appropriate transformation systems are available for various red algae and diatoms (Lapidot *et al.*, 2002; Mikami *et al.*, 2011; Karas *et al.*, 2015; Zienkiewicz *et al.*, 2019).

While it seems likely that certain PTMs found in eukaryotic red-type Rubiscos may enhance the stability of the holoenzyme, it is not known if these PTMs are essential for folding/assembly. Thus, red-type Rubisco PTM requirements may potentially be a non-trivial hurdle towards transplanting functional eukaryotic red-type Rubiscos. Consideration of green PTMs could also be vital—appending N-terminal sequence from green-type Rubisco onto introduced red-type Rubiscos may be necessary to maintain chloroplast PTMs and protect the introduced Rubisco from proteolysis, as considered in previous engineering studies (for a summary, see Sharwood, 2017). Assembly incompatibilities between tobacco and red-type Rubisco subunits (Whitney *et al.*, 2001; Lin and Hanson, 2018; Gunn *et al.*, 2020) could be advantageous as this means that green-type SSus need not be scrubbed from the nuclear genome to prevent the formation of undesirable hybrid Rubiscos. However, significant progress has been made with the capability to do so (Donovan *et al.*, 2020; Khumsupan *et al.*, 2020). To enhance red-type Rubisco activation status in chloroplasts, co-expression of a compatible CbbX is essential (Gunn *et al.*, 2020). Engineering strategies would benefit from understanding any species specificity of Rubisco–CbbX interactions, and maximal activation of Form ID Rubiscos will probably require both the nuclear- and plastid-encoded CbbX isoforms (Loganathan *et al.*, 2016; Lin and Hanson, 2018). While it is expected that the first red algal Rubisco to be successfully assembled in chloroplasts will be expressed as an operon in the chloroplast, later fine-tuning of red-type Rubisco expression could be achieved by hijacking the endogenous green SSu promoters to control total red-type Rubisco pools (Khumsupan *et al.*, 2020).

### Additional considerations for rational design approaches

Careful consideration of Rubisco evolution may aid direct Rubisco engineering strategies in approaches (i) and (ii) above. Prior success identifying catalytic switches between C_3_ and C_4_ Rubisco (Whitney *et al.*, 2011), and reconstructing ancestral Rubisco sequences with distinct catalytic signatures (Lin *et al.*, 2022) may be the tip of the iceberg in terms of how probing Rubisco evolution using phylogenetic relationships could benefit our understanding of, and ability to engineer, Rubisco. While there is potential for taking advantage of recent advances in structure prediction algorithms (Baek *et al.*, 2021; Jumper *et al.*, 2021), to make *in silico* mutations and predict their effect on structure, the relevant chemistry conferred by side chains may be beyond the current resolution limits of these approaches. This is affirmed by differences in kinetics despite relatively little structural variation in Rubiscos (Table 1; Fig. 3) and thus kinetic differences are presumably conferred by relatively subtle sequence–structural differences. Molecular dynamics simulations have contributed to our understanding of the Rubisco catalytic mechanism (Mauser *et al.*, 2001; Kannappan and Gready, 2008; Cummins *et al.*, 2019), subunit interactions (van Lun *et al.*, 2011), and the potential role of SSus as CO_2_ reservoirs (van Lun *et al.*, 2014). Further improvements to computational capabilities are exciting—especially with regards to how they could be effectively applied to the carbon fixation problem in crop species.

## Supplementary data

The following supplementary data are available at *JXB* online.

Table S1. Extended comparison table of Rubisco kinetics from red- and green-type Rubisco lineages.

erac349_suppl_supplementary_table_S1

## Data Availability

The data used in this review are all from publicly available datasets and are fully cited.

## Abbreviations

LSu: large subunit
PCA: photosynthetic carbon assimilation
RuBP: ribulose-1,5-bisphosphate
SSu: small subunit
